# Tissue microarray analyses of G1/S-regulatory proteins in ductal carcinoma *in situ* of the breast indicate that low cyclin D1 is associated with local recurrence

**DOI:** 10.1038/sj.bjc.6601398

**Published:** 2003-11-11

**Authors:** K Jirström, A Ringberg, M Fernö, L Anagnostaki, G Landberg

**Affiliations:** 1 Division of Pathology, Department of Laboratory Medicine, Lund University, Malmö University Hospital, S-205 02, Malmö, Sweden; 2 Department of Plastic and Reconstructive Surgery, Lund University, Malmö University Hospital, Sweden; 3 Department of Oncology, University Hospital Lund, Sweden

**Keywords:** Breast cancer, DCIS, cell cycle, cyclin D1, recurrence, growth pattern

## Abstract

Ductal carcinoma *in situ* (DCIS) of the breast constitutes about 10% of all diagnosed breast cancers and, despite surgical removal, it may recur, either as DCIS or invasive breast cancer. Nuclear grade and growth pattern according to Andersen *et al* as well as surgical margins are factors that have been used to predict local recurrence, but ideally a set of tumour-specific factors should be identified and used as prognostic markers. Many cell cycle regulatory gene products have been shown to be involved in the formation of tumours and are either oncogenes or suppressor genes and involved in key processes in the transformation. We therefore characterised the cell cycle regulators cyclin E, cyclin D1, p27 and p16 in a material of DCIS cases arranged in a tissue microarray. With a manual tissue arrayer, 52% of the initial 177 DCIS samples were successfully targeted allowing immunohistochemical analyses of all four proteins in 92 cases of DCIS. As also observed in invasive breast cancer, there was a trend indicating that DCIS cases with high cyclin D1 were cyclin E low and oestrogen receptor-positive, whereas cyclin E high DCIS cases were cyclin D1 low and oestrogen receptor-negative. For the 64 patients that did not receive postoperative radiotherapy, there were 16 local recurrences (eight DCIS and eight invasive breast cancer) during a mean follow-up time of 63 months. Cyclin E, p27 or p16 were not associated with local recurrence, but interestingly cyclin D1 was significantly and inversely associated with local recurrence, both using univariate and multivariate analyses. In summary, using a tissue array approach we have shown that cyclin D1, besides growth pattern, is a prognostic marker for local recurrence in DCIS.

*In situ* carcinoma of the breast has been a more frequent observation since the introduction of mammography screening, now accounting for up to 20% of all mammographically detected breast malignancies, compared to a few percent in the prescreening era (Rosner *et al*, 1980; Ringberg *et al*, 1991; Ernster *et al*, 2002) As with invasive breast cancer, noninvasive breast lesions appear to be heterogenous in nature and the relationship between preinvasive and invasive disease is not yet fully clarified. Data from histopathological studies show that proliferative changes often are found in association with invasive cancer and both atypical ductal hyperplasia (ADH) and DCIS provide a significantly increased risk of subsequent invasive carcinoma (Dupont and Page, 1985; Eusebi *et al*, 1994) Treatment with mastectomy (ME) is curative in 95–100% of all DCIS cases (Sunshine *et al*, 1985;Fentiman *et al*, 1986). Breast-conserving therapy (BCT) followed by postoperative radiotherapy (RT) provides a prognosis similar to ME (Ringberg *et al*, 2000), whereas patients having BCT without RT have an increased frequency of ipsilateral breast tumour recurrences (Ringberg *et al*, 2000).

Throughout the years, many proposals for a prognostically valuable histological classification system for DCIS have been introduced and the two most widely adopted are based on either nuclear grade (ng) in combination with architectural pattern (Holland *et al*, 1994) or the presence or abscence of comedotype necrosis (Silverstein *et al*, 1995b). Classification systems based on various differentiation aspects of DCIS seem to be clinically relevant, as the nuclear grade is strongly associated with disease recurrence (Ringberg *et al*, 2000; Boland *et al*, 2003). Unfortunately, the interobserver reproducibility of the analyses is low (Douglas-Jones *et al*, 2000; Wells *et al*, 2000) and there is still a need for a more objective basis for the classification of DCIS. Andersen *et al* (1988) have further stressed the aspect of growth pattern in DCIS . Previous investigations, where the growth pattern has been divided into diffuse (including diffuse or tumour-forming+diffuse) and nondiffuse (including microfocal or tumour forming) have also shown that a diffuse growth pattern is associated with a higher recurrence rate (Ringberg *et al*, 2000).

There is a limited amount of key events in the transformation process, and deregulation of cell cycle regulators seems to be one important and frequent finding in many cancer forms. In invasive breast cancer, many cell cycle regulators are aberrantly expressed and often linked to tumour aggressiveness and clinical outcome. Invasive breast cancers overexpressing cyclin D1 are predominantly oestrogen receptor (OR)-positive, whereas cyclin E high tumours in contrast are OR-negative and often p27 low as well as p53- and pRb-inactivated (Nielsen *et al*, 1999; Loden *et al*, 2002). It is obvious that certain genetic alterations are clustered in breast cancer and it is likely that these changes also are found in preinvasive forms of breast cancer such as DCIS.

In the present study, we wanted to determine the expression of various cell cycle regulating proteins in a series of DCIS cases arranged in a tissue microarray (TMA) and to delineate potential clusters of aberrations in cyclin D1, cyclin E, p27 and p16 and their associations to local recurrence. We further wanted to assess if TMAs are suitable for studies of preinvasive lesions such as DCIS.

## MATERIAL AND METHODS

### Patient materials

The total material consists of 177 patients with DCIS treated with BCT. The diagnoses were registered at the population-based Regional Tumour Registry in Lund between 1 September 1987 and 31 December 1991. Treatment guidelines for the Southern Health Care Region of Sweden recommended ME for DCIS lesions exceeding one fourth of the breast size or when the size or location in the breast precluded BCT with a good cosmetic outcome. Patients with smaller DCIS lesions were recommended to enter a randomised trial, studying the effect of RT after BCT. In this material, 64 patients received no adjuvant radiotherapy. The median follow-up time for the total material was 63 months and any event of ipsilateral local recurrence, DCIS as well as invasive cancer, was recorded. All tumours have earlier been reevaluated regarding histopathological features and sets of clinicopathological parameters and tumour biological factors have also been reported (OR, PgR, c-erb-B-2, bcl-2, p53, DNA ploidy status and Ki-67) (Ringberg *et al*, 2001). Ethical permission for the study was obtained from the Lund ethical committee.

### Tissue array and immunohistochemistry

Fresh slides from all blocks were reviewed for representative areas with DCIS and tissue arrays were prepared as described earlier (Kononen *et al*, 1998). In brief, two 0.6 mm punches were taken from the selected areas in each donor block and mounted in a recipient block containing approximately 200 biopsies. When neither of the two biopsies included DCIS, a new set of two biopsies were taken and mounted in a second array. For immunohistochemistry, 6 *μ*m sections of the paraffin-embedded tissue arrays were dried, deparaffinised, rehydrated and microwave-treated for 10 min in a citrate buffer (pH of 6.0) or EDTA buffer (pH of 8.0) for cyclin E before being processed in an automatic immunohistochemistry staining machine according to standard procedures (Ventana 320–202, Ventana inc., Tucson, AZ, USA or using Techmate 500, Dako, Copenhagen, Denmark). The following antibodies were used: Cyclin D1 (1 : 1000 M1755, Dako, Denmark), Ki-67, (1 : 200, M0722, DAKO, Denmark), cyclin E (1 : 100 HE12, Santa Cruz, CA, USA), p16 (1 : 200, BD PharMingen, San Jose, CA, USA), p27^Kip 1^ antibody (1 : 200, DAKO, Denmark). Antibody binding was visualised using diaminobenzidine as the chromagen and slides were counterstained with haematoxylin.

When evaluating the expression of cell cycle regulators in the DCIS arrays, the fraction of positively stained nuclei (NF), as well as the nuclear staining intensity (NI), were determined. For p16, there was no obvious variation in the nuclear intensities and NI was therefore not evaluated for p16. Due to the occasional presence of distinct cytoplasmatic staining for p16, p27 and cyclin D1, the cytoplasmic intensity (CI) was also assessed for these proteins. Both nuclear and cytoplasmic intensity were evaluated with a semiquantitative scoring system (0–3) representing, 0=none, 1=weak, 2=moderate and 3=strong, staining.

### Statistical methods

Spearman's correlation test was used for the comparison of the different parameters, whereas Kaplan–Meier and log-rank tests were used for the ipsilateral local recurrence analyses. Multivariate analyses were performed by a Cox-regression model. All calculations were performed with SPSS 11.0 (SPSS inc., Chicago, IL, USA).

## RESULTS

### Tissue array construction

**Figure 1:**
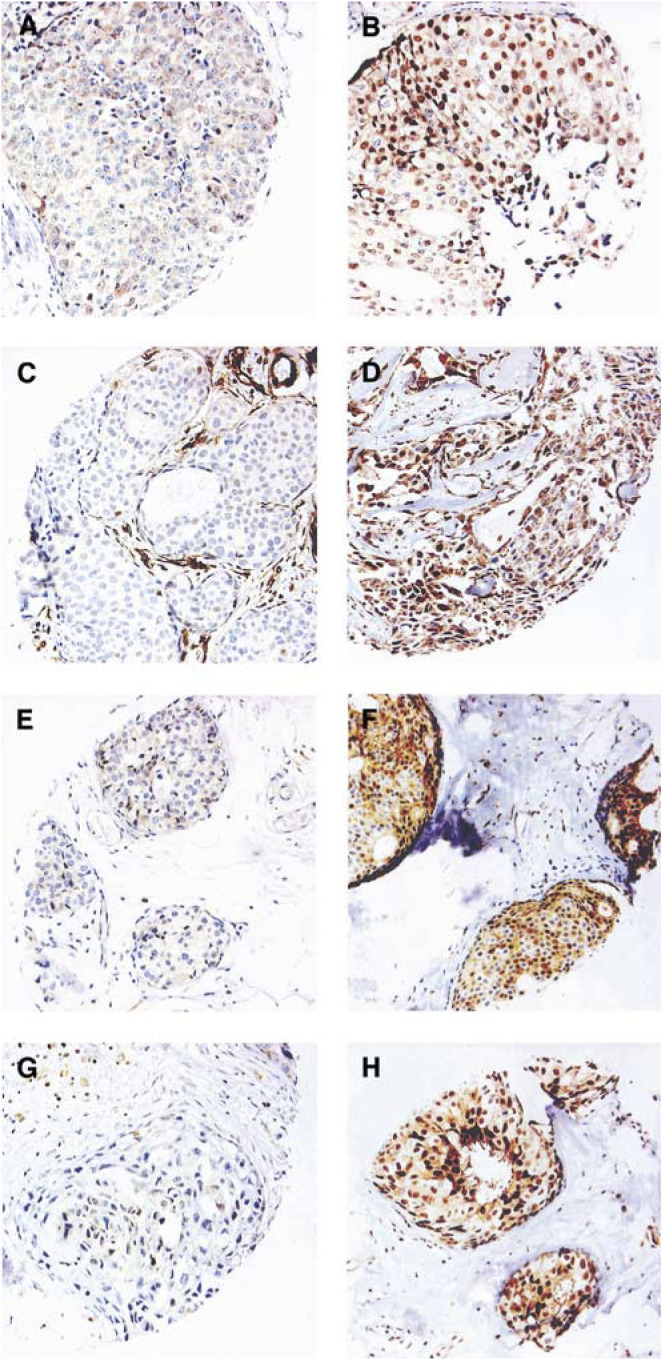
Examples of immunohistochemical staining of the cell cycle regulatory proteins in different DCIS tumours: (**A**) cyclin D1 low; (**B**) cyclin D1 high. (Note that the intensity of the cytoplasmic staining only differs slightly between (**A**) and (**B**), in contrast to the nuclear staining.); (**C**) cyclin E low; (**D**) cyclin E high; (**E**) p16 low; (**F**) p16 high; (**G**) p27 low; (**H**) p27 high.

In the present study, we wanted to test the applicability of TMAs for targeting DCIS lesions. In 92 cases (52%) out of the initial 177, we obtained relevant tissue cores with acceptable immunohistochemistry for all four cell cycle regulators as exemplified in Figure 1A

. The majority of acceptable cores were targeted in the first array. This was probably due to the fact that all blocks already had undergone extensive sectioning and, consequently, the remaining amount of representative DCIS lesions had decreased to a minimum, especially in cases with small original lesions . As expected, the precision rate depended on the size of the involved ducts *per se* as well as on the total extent of the lesion.

### Histopathological characteristics

In all, 11 of the 92 DCIS cases (11.9%) were ng 1, 37 (40.2%) ng 2 and 44 (47.8%) were ng 3. A similar distribution of the ng was seen in the remaining, unsuccessfully biopsied, DCIS, suggesting that the 92 cases were representative for a general DCIS population without any obvious selection biases (data not shown). As reported earlier, the growth pattern of DCIS might be an important prognostic factor for ipsilateral local recurrence (Ringberg *et al*, 2000) and when classified according to Andersen *et al*, the growth pattern of the DCIS cases were: 16 cases microfocal (focus⩽5 mm, no stromal reaction), 33 tumour-forming (macroscopic lesion>5 mm with stromal reaction), 37 diffuse (+/− macroscopically identifiable lesion, microscopically confluent +/− stromal reaction) and six tumour-forming and diffuse growth pattern.

In line with earlier studies, the growth pattern was divided into *diffuse* (diffuse or diffuse and tumour forming) and *nondiffuse* (tumour forming and/or microfocal) for further statistical analyses. Thus, 43 cases (46.7%) had a diffuse and 49 (53.3%) a nondiffuse growth pattern.

### Immunohistochemical detection of p16, p27, cyclin D1 and cyclin E

**Figure 2:**
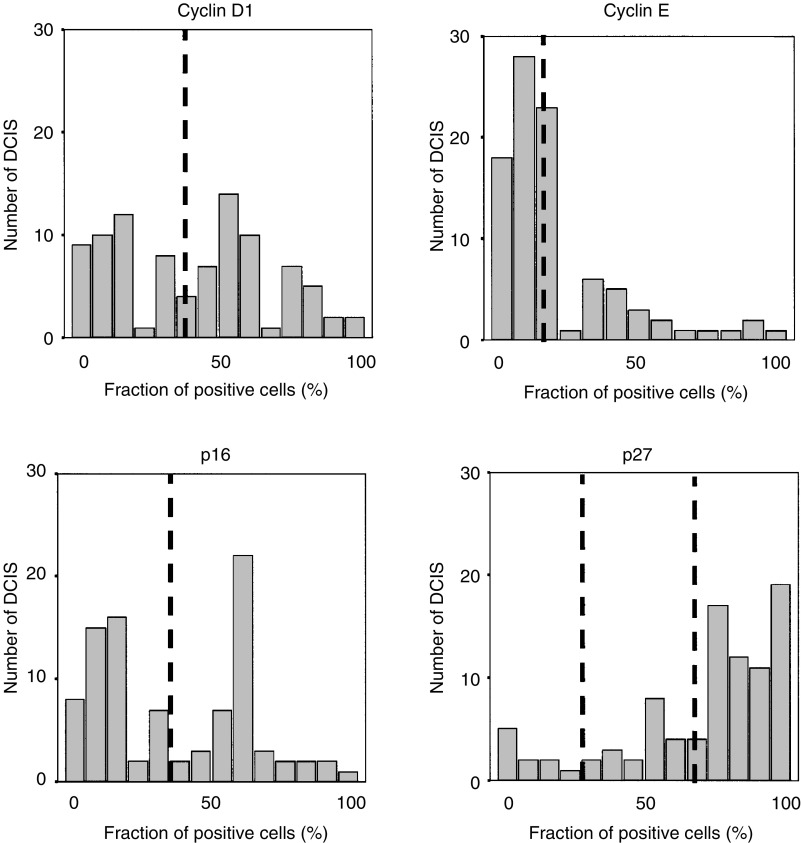
Distribution of the percentage positive cells for the analysed cell cycle regulators in 92 DCIS cases analysed by immunohistochemistry. The cutoffs used in the analyses are indicated in the figure.

In an attempt to define the existence of G1/S aberrations and possible clusters in DCIS, we analysed the protein content of several cell cycle regulatory proteins. Examples of cyclin D1, cyclin E, p16 and p27 immunohistochemical stainings are shown in Figure 1B–E and the fractions of positive cells are illustrated in Figure 2

. Focus was set on the fraction of positive cells, but similar results were obtained using staining intensities (data not shown). In short, p16 seemed to be present in various fractions in the DCIS cases and a mean value was used to split the DCIS cases in two almost equally sized groups. Using a similar approach for cyclin E, approximately 25% of the DCIS cases had high cyclin E protein expression, which also corresponded to the separately clustered tumours as denoted from the distribution illustrated in Figure 2. The majority of DCIS cases were positive for p27 and tumours were divided into three groups based on the distribution of p27 positivity (illustrated in Figure 2) producing a large group of p27-positive tumours (68.5%) and two separate groups with clear downregulation of p27. Cyclin D1 varied substantially between DCIS cases with an apparently bipolar distribution as illustrated in Figure 2, and by using the mean (37%) as cutoff, two equally sized groups were formed representative for low and high cyclin D1. Similar results were obtained when the median value (40%) for cyclin D1 was used as cutoff.

### Proliferation

Cell cycle aberrations will presumably increase the proliferation in tumours and we therefore characterised the proliferation in the DCIS material using Ki-67 as a marker. Proliferation was positively correlated to nuclear grade (*P*=0.047) and among the cell-cycle-related proteins only cyclin E correlated (positively) to proliferation (*P*=0.046).

### Associations between cell cycle regulatory proteins

Like others, we have earlier observed striking associations between various aberrations in cell cycle regulatory proteins in invasive breast carcinomas (Nielsen *et al*, 1999). In this material of DCIS, we therefore wanted to clarify any potential links between the fractions of positive cells of the analysed cell cycle regulatory proteins as well as examine their distribution and relation in scatter plots in order to reveal potential patterns formed when combining the cell cycle analyses. The associations between the cell cycle regulatory proteins are presented in Table 1

**Table 1:** Correlation analyses of cell cycle regulators and clinicopathological parameters in 92 cases of DCIS

	**Cyclin D1**	**Cyclin E**	**p16**	**p27**
Cyclin D1	—	0.257/0.119	0.0001/0.382***	0.0001/0.373***
Cyclin E	—	—	0.157/0.149	0.769/0.031
p16	—	—	—	0.039/0.215*
Ki-67	0.079/0.206	0.046/0.233*	0.545/0.071	0.451/−0.089
Age	0.486/0.074	0.592/0.057	0.784/0.029	0.048/−0.207*
OR+/−	0.114/0.203	0.312/−0.131	0.983/−0.013	0.381/0.113
PR+/−	0.171/0.147	0.098/−0.177	0.315/−0.108	0.8860/0.016
Nuclear grade	0.882/0.024	0.497/0.072	0.790/−0.028	0.886/0.016
Growth pattern	0.555/0.062	0.508/0.070	0.122/0.162	0.748/0.034

(*P*-value/*r*-value).

* *P*<0.05,

*** *P*<0.001. Progesteron receptor (PR).

. The fraction of p16-positive cells was associated with both p27 and cyclin D1 in the DCIS material. The cdk-inhibitor p27 was further strongly associated with cyclin D1 as reported earlier (Oh *et al*, 2001).

**Figure 3:**
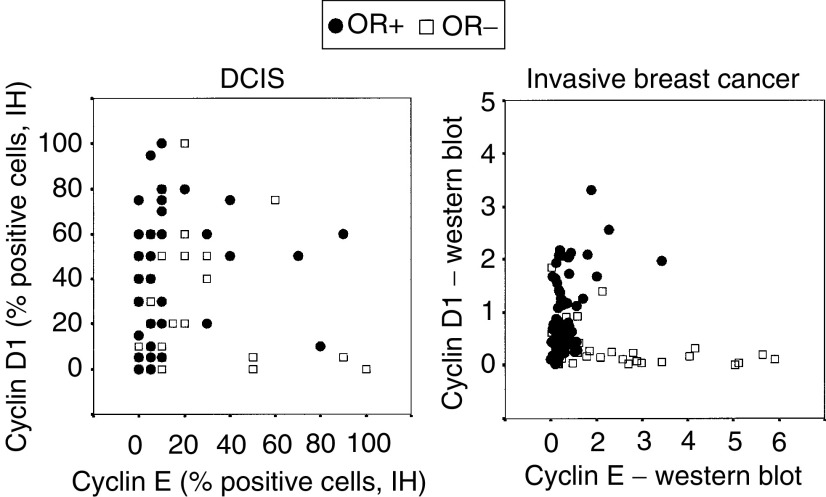
Scatter plots showing the associations between cyclin E, cyclin D1 and the OR content in DCIS and in invasive breast cancer.

Interestingly, when the fractions of cyclin D1- and cyclin E-positive cells were plotted in a scatter plot, a specific pattern was formed as illustrated in Figure 3A

. As also shown in Figure 3B, this pattern is similar to previous data obtained from Western blot analyses of invasive breast carcinomas (Nielsen *et al*, 1997), suggesting that the relation between aberrations in these two cell cycle regulatory proteins is comparable in DCIS and invasive carcinomas. The OR-content is also illustrated in Figure 3A and, noticeably, DCIS with higher cyclin D1 content were more often OR-positive, whereas tumours with high cyclin E and low cyclin D1 in general were OR-negative. This association was nevertheless not significant, probably due to the low numbers of cyclin E and D1 high DCIS.

### Cell cycle aberrations in relation to clinicopathological parameters and local recurrence

Local recurrence is a rare event after postoperative radiotherapy. In this material, 28 patients (30%) received postoperative radiation, while 64 patients (70%) did not. The overall recurrence rate in the material was 17 cases (nine DCIS and eight invasive carcinoma) and the vast majority occurred in the nonirradiated group, *n*=16. Only one local recurrence (DCIS) was seen in the group that received adjuvant radiotherapy. Thus, when associating ipsilateral local recurrence with other parameters, only the 64 cases that did not receive adjuvant radiotherapy were included. In this group, 30 cases had a diffuse and 34 cases a nondiffuse growth pattern. In total, six cases (9 %) were nuclear grade 1, 26 cases (41%) ng 2 and 32 cases (50%) ng 3. Only ipsilateral local recurrences were considered in this study and, due to the relatively small number of events, all recurrences (DCIS and invasive cancer) were reported together.

**Figure 4:**
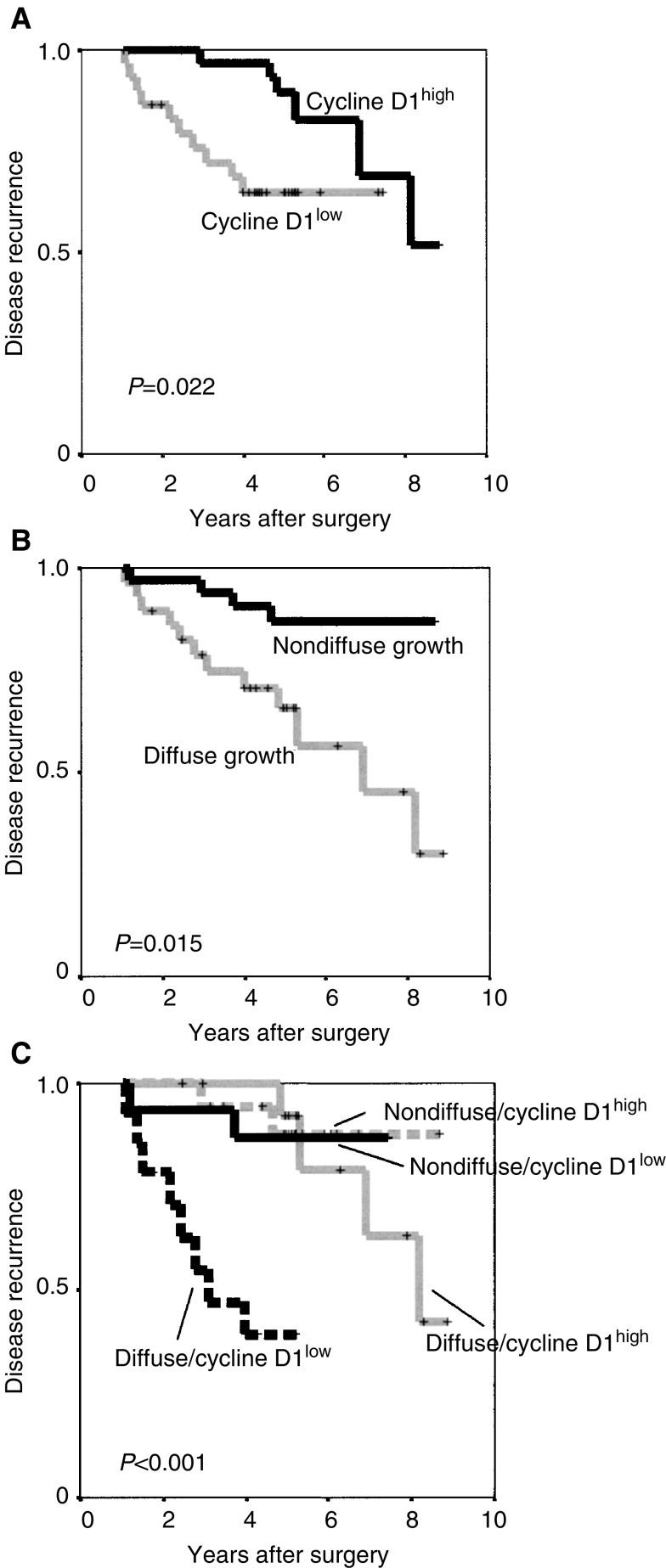
Kaplan–Meier plots of ipsilateral local recurrence (DCIS and invasive breast cancer) in the material of 64 DCIS divided according to (**A**) cyclin D1 protein content, (**B**) growth pattern according to Andersen *et al* (1988) and (**C**) a combination of cyclin D1 and growth pattern.

All data concerning potential associations between cell cycle aberrations and clinicopathological parameters are summarised in Table 1. There was no association between the analysed cell cycle regulatory proteins and growth pattern. Regarding ipsilateral local recurrence, there was no association for the cell cycle regulators, cyclin E, p16 and p27 (data not shown). Interestingly though, cyclin D1 was strongly and inversely associated with ipsilateral local recurrence as illustrated in Figure 4A

. This was true for the nuclear fraction (*P*=0.02) as well as for the cytoplasmic intensity (*P*=0.02), but not for the nuclear intensity (*P*=0.35). As shown in Table 2

**Table 2:** Fraction of disease recurrence and time to recurrence in cyclin D1 high and low DCIS (*n*=64) cases.

		**Time to recurrence**	
	**Disease recurrence (%)**	**All patients**	**Patients with recurrence**
Cyclin D1 low	32.3	4.27 years (*P*=0.001)	2.29 years (*P*=0.0002)
Cyclin D1 high	18.2	5.18 years	5.04 years

The significance values using nonparametric Mann–Whitney tests, for cyclin D1 low and high DCIS cases regarding time to recurrence, are indicated.

, the time to the first recurrence was significantly prolonged for cyclin D1 high cases. There were also fewer recurrences (six out of 33 cases=18%) in the cyclin D1 high group, compared to the cyclin D1 low group (10 out of 31 cases=32%), although this difference was not statistically significant.

Of the diffusely growing DCIS cases, 12 out of 30 (40%) had ipsilateral local recurrence compared to 4 out of 34 cases (12%) in the group with a nondiffuse growth pattern (Figure 4B). Nuclear grade was also associated to a prolonged ipsilateral recurrence-free interval (*P*=0.04). There were no local recurrences for the six six cases with nuclear grade 1, whereas four out of 26 (15%) with ng 2 and 12 out of 32 (37%) with ng 3 had local recurrences. The presence or absence of comedo-type necrosis was not significantly associated to local recurrence in the subset of 64 DCIS with available data (*P*=0.29). Margin width and tumour size were only assessed in a small subset of the included DCIS cases and therefore not further evaluated. An inclusion criterion for the study was nevertheless that the DCIS lesion should be limited and the tumour sizes of the DCIS cases in the study were presumably smaller than an unselected DCIS cohort.

In multivariate analyses, including nuclear grade, OR receptor status, growth pattern and cyclin D1, only growth pattern and cyclin D1 were significantly associated with local recurrence (Table 3

**Table 3:** Multivariate Cox-regression analyses of disease recurrence in a material of 64 DCIS cases.

			**95.0% CI**	
	**Significance**	**Exp (B)**	**Low**	**High**
Cyclin D1 (low/high)	*P*=0.007	0.146	0.036	0.589
Growth pattern (diffuse/nondiffuse)	*P*=0.007	7.180	1.713	30.095
Nuclear grade (3 *vs* 2)	*P*=0.166	2.782	0.654	11.843
(2 *vs* 1)	*P*=0.145	0.243	0.036	1.631
Oestrogen receptor	*P*=0.413	0.612	0.189	1.985

). Surprisingly, in this model, both nuclear grade and OR status were not significantly associated with local recurrence. Since both cyclin D1 and growth pattern seemed to give independent prognostic information, we also constructed a combined variable consisting of the cyclin D1 protein expression and growth pattern. As illustrated in Figure 4C, this analysis produced a highly significant difference in ipsilateral local recurrence-free interval (*p*<0.001). DCIS cases with low cyclin D1 in combination with a diffuse growth pattern had the shortest ipsilateral local recurrence-free survival and also the highest local recurrence rate with eight out of 15 cases (53%). The figure further illustrates that for the cases with a nondiffuse growth pattern, cyclin D1 protein expression only seemed to have a marginal effect on the ipsilateral local recurrence-free interval (*P*=0.83) as well as the local recurrence rate, with two out of 18 recurrences (11%) for cyclin D1 high and 2/16 (12%) for cyclin D1 low cases. For the cases with a diffuse growth pattern in combination with high cyclin D1, the local recurrence rate was four out of 16 cases (26.6%), but the ipsilateral local recurrence-free interval was significantly longer than for the cyclin D1 low cases (*P*=0.0007).

In a similar way, a combined cyclin D1 and nuclear grade variable, produced a significant difference in the ipsilateral local recurrence-free interval (*P*=0.048).

## DISCUSSION

In this study, we have observed a strong relationship between high cyclin D1 expression in DCIS lesions and a prolonged ipsilateral local recurrence-free interval, independently of the established and prognostic important clinicopathological parameter nuclear grade as well as the potentially valid prognostic factor growth pattern (Ringberg *et al*, 2000). Both the fractions of positive nuclei and the cytoplasmic intensity of cyclin D1 were significantly and inversely associated with ipsilateral local recurrence, strengthening this hypothesis. In order to increase the statistical power of the study, all ipsilateral local recurrences (DCIS and invasive cancer) were reported together. However, when local recurrences were split into DCIS and invasive breast cancer, similar Kaplan–Meier curves for the separate entities regarding cyclin D1 were produced (data not shown), but with less statistical significance (*P*=0.034 for DCIS and *P*=0.28 for invasive breast cancer). Larger studies are needed in order to more precisely delineate differences regarding cyclin D1 in DCIS and type of local recurrence.

Cyclin D1 is an important cell cycle regulating protein functioning as a regulatory subunit for two cyclin-dependent kinases, cdk 4 and cdk 6. The activated cyclin D1–cdk4/6 complexes initiate phosphorylation of pRb starting the cascade of events that lead to DNA replication and subsequent cell division (Sherr, 1996). Independently of its cdk-activating function, cyclin D1 also interacts directly with the OR, thus enhancing the transcription of OR-responsive elements (Zwijsen *et al*, 1997; Lamb *et al*, 2000). The exact mechanisms for the transactivational properties of cyclin D1, as well as the phenotypic consequences, still have to be elucidated. This may, nevertheless, be an important mechanism behind oestrogen-independent oncogenesis and for tamoxifen resistance in OR-positive breast cancers. Another interesting function for cyclin D1 is the apoptosis-stimulating properties that have been observed *in vitro* (Han *et al*, 1996; Pardo *et al*, 1996; Zhou *et al*, 2001; Katayama *et al*, 2003). In cyclin D1-overexpressing cell lines, an increased incidence of apoptosis following ionising radiation has been reported (Pardo *et al*, 1996; Zhou *et al*, 2000). Turner *et al* (2000) also provide *in vivo* evidence for a better response to RT in early-stage invasive breast cancer with high cyclin D1 levels. Further studies are nevertheless needed to clarify the role for cyclin D1 regarding an apoptotic phenotype in DCIS.

Overexpression of cyclin D1 is observed in a fraction of cases with DCIS and invasive breast cancer, and when studying precancerous forms of breast cancer the breakpoint for increased cyclin D1 expression has been observed between ADH and DCIS (Alle *et al*, 1998; Gillett *et al*, 1998). This suggests that cyclin D1 is involved in the early phase of the transformation process as also supported by findings from transgenic mice overexpressing cyclin D1 (Sutherland and Musgrove, 2002). Cyclin D1 is also essential for the formation of functional breast tissue and cyclin D1 knockout mice show defects in the formation of breast lobules during pregnancy (Fantl *et al*, 1999). The results showing similar patterns of cyclin D1 and cyclin E expression in DCIS and invasive breast cancer (Figure 3A, B) also support that DCIS is rather similar to invasive breast cancer, and that the majority of genetic aberrations in tumour progression occur at or before the stage of DCIS.

Most studies delineating the prognostic importance for cyclin D1 overexpression in breast cancer have been performed using invasive tumours and the results are diverging: some studies indicate that cyclin D1 overexpression is associated with a negative clinical outcome (Ohta *et al*, 1997; Kenny *et al*, 1999), some show no prognostic significance (Michalides *et al*, 1996; van Diest *et al*, 1997), while others report a better prognosis for cyclin D1 high tumours (Gillett *et al*, 1996; Hwang *et al*, 2003). One explanation for the discrepancy could be that the fraction of patients treated with antioestrogens varied between different studies and recent experimental findings (Bindels *et al*, 2002; Hui *et al*, 2002) suggest that cyclin D1 overexpression could induce resistance to antioestrogen treatment. Data from our laboratory on patients with invasive breast cancer, randomised to either tamoxifen or no adjuvant therapy after surgery, also support the theory that cyclin D1 overexpression is associated with an impaired prognosis in antioestrogen-treated patients, but in contrast associated with a more favourable outcome in untreated patients (to be published). The observation that cyclin D1 overexpression is associated with a prolonged ipsilateral local recurrence-free interval in cases with DCIS is therefore in line with the above-described findings. The DCIS patients in this study did not receive antioestrogen treatment after surgery.

A second objective of this study was to evaluate whether DCIS lesions are suitable for the construction of TMAs using a manual tissue array approach. Compared to invasive cancer, premalignant lesions of the breast are much more difficult to target than invasive cancer, due to the often smaller size of the lesions and more scattered appearances. Thus, the target rate of the DCIS lesions was accordingly rather low and only 52% of the DCIS cases were successfully arrayed. The paraffin blocks used for this study had also been extensively sectioned earlier and the target rate would probably increase if new paraffin blocks were used. Furthermore, the precision in targeting specific areas of a small tumour would most likely be improved if a video-equipped robotised tissue array system could be used, simultaneously visualising the donor block and the haematoxylin-stained section. Future studies will reveal the target efficiency for modern tissue arrayers.

In summary, we have demonstrated that aberrations in G1/S-regulatory proteins are present in DCIS and that they seem to be rather similar to the aberrations observed in invasive breast cancer. Among the analysed G1/S-regulatory proteins in DCIS, cyclin D1 was inversely associated to ipsilateral local recurrence. Our data clearly motivate further studies of cyclin D1 in DCIS in order to validate the importance of this highly relevant tumour biological parameter as a potential prognostic marker. As also shown, TMAs can be used for studies of DCIS, enabling high throughput analyses of preinvasive lesions, but with a rather low targeting rate using a manual tissue arrayer.
